# A Case of IL-7R Deficiency Caused by a Novel Synonymous Mutation and Implications for Mutation Screening in SCID Diagnosis

**DOI:** 10.3389/fimmu.2016.00443

**Published:** 2016-10-27

**Authors:** Fernando Gallego-Bustos, Valer Gotea, José T. Ramos-Amador, Rebeca Rodríguez-Pena, Juana Gil-Herrera, Ana Sastre, Aitor Delmiro, Ghadi Rai, Laura Elnitski, Luis I. González-Granado, Luis M. Allende

**Affiliations:** ^1^Servicio de Inmunología, Hospital Universitario 12 de Octubre, Madrid, Spain; ^2^Translational and Functional Genomics Branch, National Human Genome Research Institute, NIH, Rockville, MD, USA; ^3^Servicio de Pediatría, Hospital Universitario Clínico, Madrid, Spain; ^4^Unidad de Inmunología, Hospital Universitario La Paz, Madrid, Spain; ^5^Servicio de Inmunología, Hospital Universitario e Instituto de Investigación Sanitaria Gregorio Marañón, Madrid, Spain; ^6^Servicio de Hematología Oncología, Hospital Universitario La Paz, Madrid, Spain; ^7^Instituto de Investigación I+12, Madrid, Spain; ^8^GMGF, Aix-Marseille Université, Marseille, France; ^9^UMR_S 910, INSERM, Marseille, France; ^10^Unidad de Inmunodeficiencias, Pediatría, Hospital Universitario 12 de Octubre, Madrid, Spain

**Keywords:** IL-7R, primary immunodeficiency, SCID, splicing, synonymous substitutions

## Abstract

Reported synonymous substitutions are generally non-pathogenic, and rare pathogenic synonymous variants may be disregarded unless there is a high index of suspicion. In a case of IL7 receptor deficiency severe combined immunodeficiency (SCID), the relevance of a non-reported synonymous variant was only suspected through the use of additional *in silico* computational tools, which focused on the impact of mutations on gene splicing. The pathogenic nature of the variant was confirmed using experimental validation of the effect on mRNA splicing and IL7 pathway function. This case reinforces the need to use additional experimental methods to establish the functional impact of specific mutations, in particular for cases such as SCID where prompt diagnosis can greatly impact on diagnosis, treatment, and survival.

## Introduction

**Figure 1:**
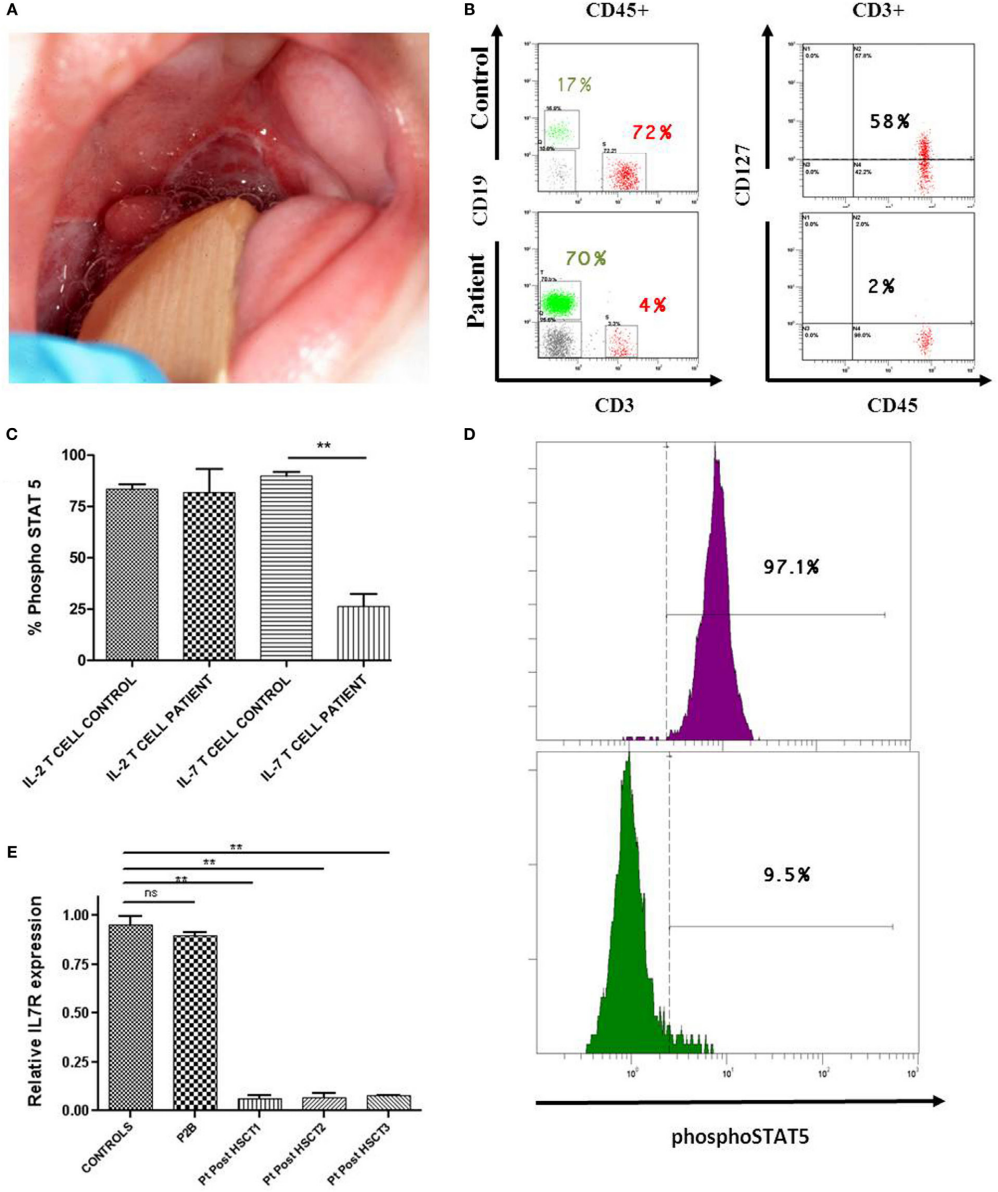
**Immune evaluation of patient deficient in IL-7R**. **(A)** Two major aphthous oral ulcers (large ulcers greater than 10 mm in diameter) in a 5-month-old male, involving the posterior veil of soft palate. **(B)** Lower CD127 expression on CD3 T-cells of IL-7R-deficient patient compared with a healthy donor. **(C,D)** Significantly lower phosphorylation of STAT5 (phosphoSTAT5, BD biosciences) in response to IL-7 (R&D systems) was observed in T-cells from the patient post-HSCT (IL-7 T cell patient) compared with the healthy donor (IL-7 T cell control). An example of phosphorylation of STAT5 with IL-7 in T-cells from the control (in purple) vs. phosphorylation of STAT5 with IL-7 in T-cells from the patient post-HSCT (in green). Normal phosphoSTAT5 in response to IL-2 (Roche) was observed in the patient (IL-2 T cell patient) and the healthy donor (IL-2 T cell control) (*n* = 2). Statistical comparison was performed with unpaired Student’s *t*-tests, with significance defined as **P < 0.01; ns: not statistically significant. **(E)** Impaired *IL7R* gene expression in CD3 T-cells from IL-7R-deficient patient 1, 2, and 3 years post-HSCT (Pt post-HSCT 1, 2, and 3). These results were compared with P2B (IL-7R-deficient patient with complete immune reconstitution after 19 years post-HSCT) and 10 healthy children donors. GADPH was used as the endogenous gene control. Triplicate data from one experiment are shown.

**Figure 2:**
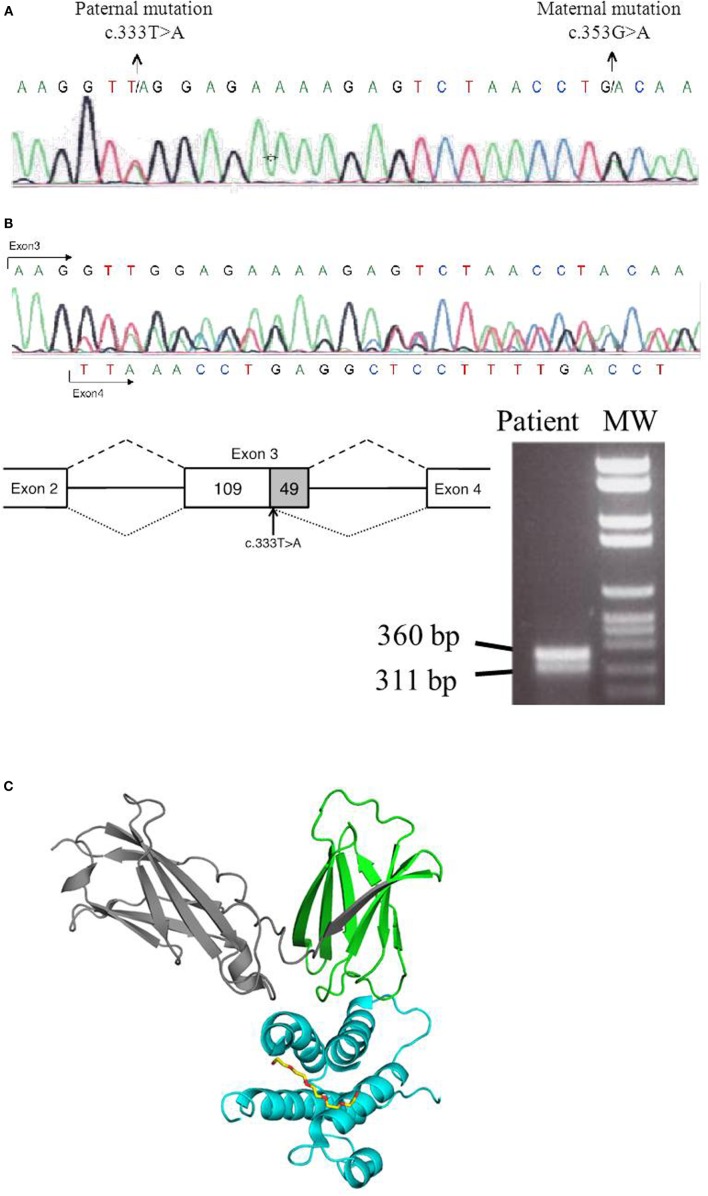
**Genetic analysis of *IL7R* gene**. **(A)** *IL7R* genomic DNA sequence showing c.333T>A and c.353G>A mutations. **(B)** Representation of the exon 3 truncation in the paternal mutated allele (c.333T>A), in which the last 49 nucleotides of exon 3 are skipped. cDNA was obtained from the IL-7R-deficient patient, and RT-PCR was done using following set of primers corresponding to *IL7R* that amplified a part of exon 2, exon 3, and a part of exon 4 (IL7R-Exon2-F-5′-GACCTGTGCTTTTGAGGACC and IL7R-Exon4-R-5′-TCATCCTTTTCCTGGCGGTA). RT-PCR gel shows two bands corresponding to maternal allele (360 bp) and the paternal allele (311 bp) that lacked the last 49 nucleotides of exon 3. Sequencing trace of the *IL7R* cDNA sequence illustrates the presence of the truncated version, as well as the presence of the maternal allele in the non-truncated version of the mature mRNA. **(C)** Structural analysis of IL7R–IL7 complex was done using the structure prediction of IL7R–IL7 as reference PDB code: 3UP1 (2). The C-terminus IL-7R protein that is truncated after residue 119 is colored in gray. Structure image was done with the Pymol software package (PyMOL Molecular Graphics System).

We report a 5-month-old male born to non-consanguineous parents who fulfilled clinical and immunological parameters for severe combined immunodeficiency (SCID). He was admitted with a history of high fever, diarrhea, and oral thrush (Figure 1A). Physical examination revealed failure to thrive and diaper candidiasis. Immunophenotyping showed T-cell lymphopenia and normal to elevated B and NK cells. The lymphoproliferative response was absent, and serum immunoglobulins were very low, leading to the suspicion of T^−^B^+^NK^+^ SCID (Table 1). First, mutations in the *IL2RG* gene were ruled out, and autosomal recessive IL-7R deficiency was suspected. Sanger sequencing of the *IL7R* gene revealed a heterozygous non-synonymous mutation (c.353G>A, p.C118Y) in exon 3 (Figure 2A). This mutation, inherited from his healthy mother, was previously reported in SCID patients (1). No other missense and nonsense mutations, as well as insertions and deletions, were found in coding exons or exon–intron boundaries in genomic DNA. Since the phenotype of the patient clearly pointed to an IL-7R deficiency due to the absence of CD127 expression and naive CD4 and CD8 T-cells (Table 1; Figure 1B), we revaluated the coding sequence of the *IL7R* gene. Apart from the c.353G>A mutation, we only found another heterozygous synonymous mutation that affects codon 111 (c.333T>A, p.V111V) (Genbank NM_002185.3). This mutation was inherited from his healthy father, but its absence from various mutation repositories (dbSNP v.146, Human Gene Mutation Database Professional, ClinVar, 1000 Genomes Project) indicates that it is a rare polymorphism. The CADD tool (http://cadd.gs.washington.edu/score), which was developed to evaluate the deleteriousness of various mutation types, indicated a low potential impact of this mutation (raw score of −0.077; PHRED-like scaled score of 1.906). However, we also evaluated the potential functional impact of this mutation by evaluating its influence on gene splicing with computational tools developed specifically for this purpose (for materials, see Supplementary Material). SplicePort and MaxEntScan indicated that a cryptic donor splice site just upstream of this mutation could be activated, which would result in the truncation of the last 49 nucleotides of exon 3. To verify this prediction, we analyzed the splicing pattern of *IL7R* mRNA extracted from patient’s PBMCs. In agreement with the expectations, the RT-polymerase chain reaction (PCR) gel revealed the presence of two bands (Figure 2B). Sequencing of RT-PCR products confirmed that the larger fragment contained all correctly spliced exons, whereas the smaller fragment lacked the last 49 nucleotides of exon 3 (Figure 2B). This deletion truncation results in a frameshift that leads to a premature stop codon at residue 119 (c.330del49) and truncation of the protein C-terminus (Figure 2C), indicating loss of functional receptors.

**Table 1:** **Immunological features of the patient**.

Parameter	Normal range	Pre-HSCT	1 year after HSCT	2 years after HSCT	3 years after HSCT	P2B
**Lymphocyte count (no./μL) and phenotype (%)**	2500–6000	2970	410	1951	1696	1580
**T cells**						
CD3^+^ (%)	52–88	6	89	38	59	75
CD3^+^CD127^+^ (%)	50–85	2	n.d.	26	13	70.6
CD4^+^ (%)	33–55	3	23	10	20	39
CD4^+^CD45RA^+^CCR7^+^ (%)	48–72	0	0	11	5.1	22.2
CD4^+^CD45RA^−^CCR7^+/−^ (%)	22–42	99	99	83	94	78
CD4^+^CD45RA^+^CD31^+^ (%)	44–60	n.d.	n.d.	7	4	17.5
CD8^+^ (%)	17–34	2	70	26	36	35
CD8^+^CD45RA^+^CCR7^+^ (%)	28–61	0	14	3.5	1.3	46.6
CD8^+^CD45RA^−^CCR7^+^ (%)	2.9–7	99	84	5.7	6.4	31.6
CD8^+^CD45RA^−^CCR7^−^ (%)	19–35			55.7	85.2	20.1
CD8^+^CD45RA^+^CCR7^−^ (%)	9–34			35.1	7.1	1.7
CD8^+^CD57^+^ (%)	1–20	n.d.	n.d.	57.1	68.4	3
**B cells**						
CD19^+^ (%)	9–28	66	0^a^	40.7	30.7	15.3
CD19^+^CD27^+^ (%)	12–35	n.d.	n.d.	5.8	n.d.	34.8
CD19^+^IgD^+^CD27^−^ (naive) (%)	57–84	n.d.	n.d.	90.7	n.d.	56.3
CD19^+^IgD^+^CD27^+^ (marginal) (%)	4–12	n.d.	n.d.	1.6	n.d.	11.1
CD19^+^IgD^−^CD27^+^ (switched) (%)	7–25	n.d.	n.d.	4.2	n.d.	23.5
CD19^+^CD21^low^ (%)	3.5–12.5	n.d.	n.d.	13.1	n.d.	2.1
**NK cells**						
CD56^+^CD3^−^ (%)	2–20	23	10	21.2	9.7	6.3
**Lymphoproliferation (cpm)**						
PHA	>50,000	690	11,582	38,610	25,404	140,866
Anti-CD3	>20,000	497	4587	332	900	48,956
Anti-CD3 + anti-CD28	>50,000	583	7589	10,348	28,770	153,308
**Serum immunoglobulins**						
IgG (mg/dL)	400–1100	232	n.d.	1120	649	1090
IgA (mg/dL)	10–160	<6.67	n.d.	312	341	255
IgM (mg/dL)	50–180	29	n.d.	133	66	61
IgE (IU/mL)	5–165	<5	n.d.	5	n.d.	n.d.

*^a^Rituximab*.

*n.d., not determined*.

Once the IL-7R deficiency was confirmed, the decision to offer hematopoietic stem cell transplantation (HSCT) was made. The patient received HSCT from an unrelated HLA identical donor at the age of 10 months. The conditioning regimen included ATG, fludarabine, and melphalan. He achieved full donor chimerism 2 months after HSCT (Table S1 in Supplementary Material). The patient is currently 4 years old and although successfully rescued from SCID by HSCT, he has suffered severe complications: hepatic graft-versus-host disease (GvHD), severe malnutrition, and diarrhea caused by norovirus infection, respiratory infections caused by RSV and EBV, steroid-resistant chronic autoimmune thrombocytopenia (<10,000/mm^3^) requiring Rituximab, *Klebsiella oxytoca*, and *Enterobacter cloacae* sepsis that needed vasoactive drugs and pediatric intensive care unit admission. Immunological reconstitution of the patient was deficient in the T-cell compartment due to reduced recent thymic emigrants, decreased naive CD4 and CD8 T-cells with a senescent T-cell phenotype, reduced CD127 expression, and impaired proliferation to phytohemagglutinin (PHA) stimulation (Table 1). Analysis of IL-7 induced pSTAT5 (Y694) in T-cells from the patient was also reduced in comparison with healthy donors (Figures 1C,D). At molecular level, *IL7R* mRNA expression in T-cells in samples post-HSCT was severely reduced in the patient compared with healthy donors (Figure 1E). The immunological findings after HSCT of this patient were compared with another IL-7R deficient patient (P2B) (3) who experienced complete immune reconstitution 19 years post-HSCT (Table 1). The protocols of this study were approved by the Institutional Review Board of Hospital Universitario 12 de Octubre (Madrid, Spain).

## Methods

### Flow Cytometry

Proportions and lymphocyte count of T-, B-, and NK-cells were determined in blood samples using conjugated mouse antihuman monoclonal antibodies and analyzed by flow cytometry using a Navios Cytometer (Beckman Coulter, Madrid, Spain).

### Proliferation Assay

Heparinized whole blood from the patients were diluted in complete medium and cultured with the following mitogens: PHA (Sigma, Madrid, Spain), anti-CD3 antibody, and anti-CD3 + anti-CD28 antibodies (BDbiosciences, Madrid, Spain). The samples were cultured in triplicate wells for 3 days at 37°C in a humidified incubator containing 5% CO_2_, then the wells were pulsed individually with 1 μCi of [^3^H]-thymidine and incubated during the last 18 h. The amount of radioactivity was measured in a scintillation counter with results expressed as counts per minute.

### Immunoglobulins

Total serum immunoglobulins (IgG, IgA, IgM, and IgE) were measured by nephelometry (Beckman Coulter, Madrid, Spain).

### Molecular Genetics

Genomic DNA was extracted from EDTA whole blood using a MagNa Pure Compact Nucleic Acid Isolation Kit (Roche, Madrid, Spain). PCR to amplify the eight exons of *IL7R* gene, and their flanking regions were carried out using specific primers (Primers available upon request). Purified PCR products were sequenced using an ABI PRISM 3130 genetic analyzer.

### Gene Expression

Total RNA was isolated with the RNeasy plus mini kit (Quiagen, Madrid, Spain). One microgram of total cellular RNA was reverse transcribed using a Transcriptor First Strand cDNA Synthesis Kit (Roche). *IL7R* gene expression quantification was measured by qRT-PCR using a TaqMan probe in a LightCycler 480 instrument (Roche) according to the manufacturer’s protocol. GADPH was used as the endogenous control, and the level of expression of *IL7R* was quantitatively measured relative to that in 10 different donors.

## Background

Severe combined immunodeficiency is a life-threatening disorder characterized by profound impairment of both cellular and humoral immunity. Patients suffer severe and opportunistic infections, protracted diarrhea, and failure to thrive, leading to a fatal outcome in their first year of life (4). SCID is a genetically heterogeneous disorder caused by mutations in more than 20 different genes and is classified according to the immunological phenotype and the corresponding underlying molecular defect. T^−^B^+^NK^+^ SCID phenotype is frequently caused by defects in the α chain of the interleukin-7 receptor (IL-7R or also known as CD127) (5). More than 50 cases of IL-7R deficiency have been described (approximately 10% of SCID cases). A rapid molecular screen is essential to distinguish among SCID patients, so they undergo early HSCT (6).

## Discussion

The c.333T>A mutation illustrates the first case of a synonymous nucleotide substitution in the coding region of the *IL7R* gene that results in abnormal splicing. We note that, as expected for a fourfold degenerate site, the site of this mutation appears to have evolved essentially neutrally in the mammalian lineage as suggested by low, negative *phyloP*, and GERP++ scores (−0.1606 and −1.61, respectively). However, the *phyloP* score computed for the primate lineage (0.505) indicates a high level of conservation of this site in primates, which are the only species that have a GT dinucleotide (specific for a donor splice site) preceding it. Therefore, it is tempting to speculate that conservation of this site in primates was maintained to avoid activation of the cryptic donor splice site in a highly conserved exon (both donor and acceptor consensus dinucleotides are perfectly conserved throughout vertebrates). However, half of all 18 fourfold degenerate sites in exon 3 exhibit similar levels of high conservation in primates, indicating that other mechanisms might be at play concomitantly. This aspect is reinforced by a T>C transition reported at this site (rs199641706) but which is not predicted to activate the preceding cryptic splice site in a manner similar to c.333T>A (Table 2). Nonetheless, it is clear that impact on splicing of this exon can lead to severe phenotypes, as illustrated in this study, which supports splicing as one of the evolutionary forces that maintains the conservation of this site.

**Table 2:** ***In silico* analysis of the impact of four synonymous mutations on the strength of neighboring canonical and cryptic donor splice sites using four computational tools**.

	Features/scores	Mutation			
		c.333T>A	c.1767C>T	c.2961C>T	c.333T>C
Tool	Gene	IL7R	JAK3	JAK3	IL7R
	Chromsome	5	19	19	5
	Genomic coordinate (hg19)	35,867,519	17,947,957	17,942,054	35,867,519
	Gene strand	+	–	–	+
	Reference transcript	NM_002185	NM_000215	NM_000215	NM_002185
	Exon number	3	13	21	3
	Exon size (bps)	158	85	173	158
	Distance from donor SS	47	20	18	47
	Activated cryptic 5′ SS	35,867,516	17,947,959	17,942,056	35,867,516
SplicePort	Canonical 3′ SS	0.652895	0.870339	0.721877	0.652895
	Canonical 5′ SS	1.50677	0.446207	−1.01599	1.50677
	Cryptic 5′ SS	−0.260474	NA	NA	−0.260474
	Mutant canonical 5′ SS	1.03778	0.32365	−1.24002	1.56303
	Mutant cryptic 5′ SS	0.588026	0.634022	0.62936	−0.280607
	Δ canonical 5′ SS	−0.46899	−0.12256	−0.22403	+0.05626
	Δ cryptic 5′ SS	+0.84850	+0.634022	+0.62936	−0.02013
Human Splicing Finder	Canonical 3′ SS	90.75	85.87	88.44	90.75
	Canonical 5′ SS	95.68	80.22	83.82	95.68
	Cryptic 5′ SS	83.35	57.5	65.81	83.35
	Mutant canonical 5′ SS	95.68	80.22	83.82	95.68
	Mutant cryptic 5′ SS	88.37	84.33	92.64	83.35
	Δ canonical 5′ SS	0	0	0	0
	Δ cryptic 5′ SS	+6.02	+26.83	+26.83	0
MaxEntScan	Canonical 3′ SS	9.26	8.5	9.26	9.26
	Canonical 5′ SS	9.14	3.45	3.44	9.14
	Cryptic 5′ SS	5.64	-0.78	-0.34	5.64
	Mutant canonical 5′ SS	9.14	3.45	3.44	9.14
	Mutant cryptic 5′ SS	9.45	6.96	7.41	4.85
	Δ canonical 5′ SS	0	0	0	0
	Δ cryptic 5′ SS	+3.81	+7.74	+7.75	−0.79
NNSPLICE	Canonical 3′ SS	0.96	0.81	0.45	0.96
	Canonical 5′ SS	1.00	0.06	0.05	1.00
	Cryptic 5′ SS	0.25	NA	NA	0.25
	Mutant canonical 5′ SS	1.00	0.06	0.05	1.00
	Mutant cryptic 5′ SS	0.92	0.90	0.79	0.14
	Δ canonical 5′ SS	0	0	0	0
	Δ cryptic 5′ SS	+0.67	+0.90	+0.79	−0.11

*(i) The mutation c.333T>C in *IL7R* is included in this table to illustrate the fact that although it occurs at the same position as the c.333T>A mutation, it is predicted to have an opposite effect on neighboring donor splice sites. Specifically, instead of activating the cryptic donor (5′) splice site, it weakens it, and at the same time, it strengthens the canonical donor splice site of the exon*.

*(ii) The coordinate of the activated cryptic 5′ (donor) splice site corresponds to the last exonic nucleotide that precedes the GT consensus dinucleotide. Several putative cryptic splice sites were evaluated, but only the one with the highest mutant score is reported here*.

*∆ canonical 5′ SS in red represents a weakening of the canonical donor splice site of the exon 3*.

*∆ canonical 5′ SS in green represents a strengthening of the canonical donor splice site of the exon 3*.

*∆ cryptic 5′ SS in red represents a weakening of a cryptic donor splice site*.

*∆ cryptic 5′ SS in green represents an activating of a cryptic donor splice site*.

Synonymous mutations that affect splicing were known for decades to cause various disease phenotypes (7). In the case of SCID, only two other synonymous mutations were documented, despite the increasing number of diagnosed patients by developing newborn screening programs in Europe and USA (4). Both of those mutations are located in the *JAK3* gene: c.1767C>T in exon 13 (8) and c.2961C>T in exon 21 (9). Similarly to the case of the *IL7R* mutation investigated here, the deleterious effect of these mutations results from their activating of a cryptic donor splice site. Computational predictions of cryptic splice site activation can be made with tools such as SplicePort and MaxEntScan that can be used for the evaluation of such scenarios (Table 2; Figure S1 in Supplementary Material), although the process is not fully automated. In contrast, tools that predict splicing alteration without specifically evaluating activation of cryptic splice sites have little predicting power for mutations like the ones described herein (Table S2 in Supplementary Material), but they do remain important tools when specific mutations do not activate cryptic splice site. Since effect of mutations cannot be known *a priori*, it is always recommended that predictions are performed using multiple tools. However, experimental validation of computational predictions needs to be performed whenever you suspect a splicing defect in order to give the appropriate diagnosis.

## Concluding Remark

We report the first case of an *IL7R* causal synonymous mutation in a male patient with T^−^B^+^NK^+^ SCID phenotype. This mutation (c.333T>A), inherited from his healthy father, activates a cryptic exonic donor splice site that leads to exon truncation (r.330del49) and premature stop codon. It added to a symptomatic missense mutation (c.353G>A, p.C118Y) inherited from his healthy mother and together render both IL7R copies non-functional. This case reinforces the need for researchers to functionally evaluate all mutations found in patients. The advent of next-generation sequencing (NGS) provides to researchers with increased genomic coverage, which in turn requires increased efforts for mutation screening. The ever-increasing panel of ever-improving computational tools can facilitate such efforts of functional prioritization, especially in the case of synonymous mutations. Nonetheless, detailed functional reports of specific mutations in patients, such as our report of a causal synonymous mutation in a SCID patient, are ultimately necessary to provide a better understanding of functional impact within the context of specific disease phenotypes.

## Author Contributions

FG-B performed the laboratory work for this study, computational predictions, and drafted the manuscript. VG performed computational predictions and drafted the manuscript. JR-A, RR-P, JG-H, AS, and LG-G were responsible for the clinical management of the patients. AD, GR, and LE collaborated in computational predictions. LG-G drafted the manuscript. LA designed the research, collaborated in computational predictions, and drafted the manuscript.

## Conflict of Interest Statement

The authors declare that the research was conducted in the absence of any commercial or financial relationships that could be construed as a potential conflict of interest.

## Abbreviations

ATG, anti-thymocyte globulin; CADD, combined annotation-dependent depletion; GvHD, graft-versus-host disease; HSCT, hematopoietic stem cell transplantation; IL-7R, interleukin 7 receptor; NGS, next-generation sequencing; PBMCs, peripheral blood mononuclear cells; PHA, phytohemagglutinin; SCID, severe combined immunodeficiency; SS, splice site.
