# Supplementary material for: A Case of IL-7R Deficiency Caused by a Novel Synonymous Mutation and Implications for Mutation Screening in SCID Diagnosis
Source: Front Immunol. 2016 Oct 27;7:443. doi: 10.3389/fimmu.2016.00443 (PMC5081475; doi:10.3389/fimmu.2016.00443)
Supplement: Supplementary file 1 [file Data_Sheet_1.PDF]

Supplementary Material

A case of IL-7R deficiency caused by a novel synonymous mutation and implications for mutation screening in SCID diagnosis

Fernando Gallego-Bustos[1], Valer Gotea[2], José T. Ramos-Amador[3], Rebeca Rodríguez-Pena[4], Juana Gil-Herrera[5], Ana Sastre[6], Aitor Delmiro[7], Ghadi Rai[8,9], Laura Elnitski[2], Luis I. González-Granado[7,10], Luis M. Allende[1,7*]

[1] Servicio de Inmunología, Hospital Universitario 12 de Octubre, Madrid.
[2] Translational and Functional Genomics Branch, National Human Genome Research Institute, NIH, Rockville, MD, USA.
[3] Servicio de Pediatría, Hospital Universitario Clínico, Madrid.
[4] Unidad de Inmunología, Hospital Universitario La Paz, Madrid.
[5] Servicio de Inmunología, Hospital Universitario e Instituto de Investigación Sanitaria Gregorio Marañón, Madrid.
[6] Servicio de Hematología Oncología, Hospital Universitario La Paz, Madrid.
[7] Instituto de Investigación I+12, Madrid.

[8] Aix-Marseille Université, GMGF, Marseille, France

[9] Inserm, UMR_S 910, Marseille 13385, France

[10] Unidad de Inmunodeficiencias, Pediatría, Hospital Universitario 12 de Octubre, Madrid

***Correspondence**: Luis M. Allende. Servicio de Inmunología. Hospital Universitario 12 de Octubre. Avda. Cordoba s/n. Madrid-28041 (Spain). E-mail: luis.allende@salud.madrid.org

1 Supplementary Data

The most widely recognized effect of mutations is altering the amino acid sequence of the encoded protein. However, mutations can have more subtle effects, among which is the alteration of gene splicing patterns. Mutations can affect gene splicing mainly through altering splice site recognition sequences or exonic and intronic splicing regulatory sequences (ESRs or ISRs, respectively).

The vast majority of splice sites are recognized by the major spliceosome, and they follow specific consensus sequences (S1): YAG/guragu for the 5' (donor) splice site, and nyag/G preceded by a variable length polypyrimidine tract for 3' (acceptor) splice site (Y indicates a pyrimidine, R indicates A or G, / indicates the actual splice site, capital letters indicate exonic sequences, small letters indicate intronic sequences). Therefore, the vast majority of introns are flanked by the consensus dinucleotide sequences GT at their 5' end and AG at their 3' end. Various computational tools have been developed to score the strength of splice sites, and therefore the impact of mutations can be evaluated with the help of such tools by analyzing the change in score induced by specific mutations.

Exonic and intronic splicing regulatory sequences bind splicing activator or repressor proteins, respectively (S1). Splicing activators help recruit spliceosomal components to nearby splice sites, while splicing repressors block or inhibit the recruitment of spliceosomal molecular complex. It therefore follows that change in the RNA sequence might change the binding dynamics of splicing enhancer and silencer proteins with direct implications for the mature mRNA molecule. Certain computation tools can quantify the changes in the binding sites and predict the directionality of the change toward increased exon skipping or retention rates.

Here we use six computational tools to evaluate the predicted impact of three synonymous mutations (one in *IL7R* and two in *JAK3*) found in SCID patients on splicing of the affected genes (Tables 2 and S2). We also evaluated the impact of the c.333T>C mutation in *IL7R*, because it occurs at the same position as the c.333T>A mutation described in this study and was described previously as a polymorphism (rs199641706). As various tools use different features, algorithms and score scales, it is important to keep in mind that scores produced by different tools cannot be directly compared.

SplicePort (http://www.spliceport.org; http://spliceport.cbcb.umd.edu) uses sequence features located within 80 bps of GT or AG consensus dinucleotides to produce a splice site strength score for donor and acceptor splice sites, respectively (S2). Its web interface also allows users to explore the sequence features that contributed more heavily toward the final score. The main drawback of this tool consists of not currently having implemented an option for evaluating the effect of specific mutations, so it requires additional user effort to score specific splice sites both in the absence and presence of mutations of interest. However, it presents the advantage that it can evaluate the impact of mutations located as far as 80 bps from the consensus splice site dinucleotide. In the case of the c.333T>A mutation in *IL7R*, SplicePort is the only tool that shows this mutation to simultaneously weaken the canonical donor splice site of exon 3 and significantly strengthen the cryptic splice site located just upstream of it (Table 2). These scores allow users to predict a likely truncation of exon 3 by 49 nucleotides. Similar interpretations can be made for the two synonymous mutations in *JAK3*,

with the only difference that those mutations activate cryptic splice sites by creating the GT dinucleotides that benefit from a sequence context closely resembling splice site consensus sequences. SplicePort cannot score splice site sequence propensities in the absence of consensus dinucleotide, and thus “NA” values are shown for the wild-type cryptic splice sites. In contrast, it shows that the c.333T>C mutation in *IL7R*, has the opposite effect and therefore is unlikely to lead to exon truncation.

Human Splicing Finder (http://www.umd.be/HSF3) evaluates the strength of splice sites using 9-mer and 14-mer sequences containing donor and acceptor sites, respectively (S3). Similarly to SlicePort, one could evaluate the impact of a mutation by comparing scores in the absence and in the presence of specific mutations, but unlike SplicePort, these mutations need to be much closer to the consensus dinucleotide in order to be evaluated. Therefore, whereas activation of a nearby cryptic splice site can be highlighted by this tool, the concomitant negative impact on the canonical donor splice site cannot (Table 2). A user-friendly feature of Human Splice Finder is a data input mode that allows automated evaluation of a mutation impact. The user simply provides sequences that differ by specific mutation(s), and the tools automatically compute relevant changes induced by the presence of those mutations. It also provides graphical representation of splice site positions and scores, as well as splicing regulatory sequences (Figure S2). For example, the segment containing the c.333T>A mutation (indicated by the red oval) experiences increased ESE/ESS ratio, which might indicate increased exon retention in the presence of the mutation. However, activation of a cryptic splice site in this case takes functional precedence as shown experimentally, and therefore one has to be mindful about various splicing aspects in the final interpretation of the functional impact of specific mutations. Additionally, Human Splice Finder integrates splice site strength evaluation using the MaxEntScan algorithm (see below), and therefore it provides an additional interface for MaxEntScan with the included benefits of automated evaluation of mutation impact.

MaxEntScan (http://genes.mit.edu/burgelab/maxent/Xmaxentscan_scoreseq.html) is a computational tool that uses maximum entropy models to score the strength of splice sites (S4). Models were built with 9-mers (3 exonic and 6 intronic nucleotides) for 5’ (donor) splice sites and 23-mers (20 intronic and 3 exonic nucleotides) for 3’ (acceptor) splice sites. Similarly to the case of SplicePort, the original implementation requires additional user effort in order to evaluate the impact of specific mutations, namely scoring the wild-type and variant version of a given splice site and then computing the change in score. However, other tools, such as Human Splice Finder and Skippy, have incorporated the MaxEntScan algorithm and facilitate this analysis. We note here that MaxEntScan is the only tool that scored the cryptic splice site activated by the c.333T>A mutation in *IL7R* higher than the canonical donor splice site of exon 3, strongly suggesting a switch in splice site usage that leads to the 49-bp exon truncation (Table 2).

NNSPLICE (http://www.fruitfly.org/seq_tools/splice.html) is a tool that can evaluate strength of splice sites based on known splice sites in *Drosophila melanogaster* and human (S5). It uses improved neural network recognizers that were pioneered by Brunak *et al.* for splice site recognition (S6). Similarly to SplicePort, this tool scores only splice sites flanked by consensus GT and AG dinucleotides (for donor and acceptors splice site, respectively), and does not provide automated evaluation of mutation impact. Similarly to the other tools presented above, NNSPLICE indicates significant score increases for the cryptic splice sites neighboring the three mutations of interest (Table 2), leading for a consensus interpretation of cryptic splice site activation and exon truncation.

Skippy (http://research.nhgri.nih.gov/skippy/) is a tool that was designed to evaluate the impact of single nucleotide variants on splicing of affected exons (S7). Unlike the four tools included in Table 2, Skippy does not evaluate the strength of splice sites. Instead, it analyzes the change in the panel of exonic splicing enhancers (ESEs) and exonic splicing silencers (ESSs) induced by a given mutation (Figure S1). Through comparisons with effects induced by mutations known to lead to exon skipping and mutations not to affect splicing, this tool computed a log odds ratio (LOR) score for mutation located in internal exons (mutations in first or last exons are not evaluated). Positive LOR scores indicate an effect similar to that of splice-affecting variants, and an empirical threshold of 1.2 could be used to select variants that are likely to cause exon skipping. Values close to 0 could be interpreted as indicative of little impact on splicing. All three mutation of interest exhibit relatively low LOR scores (Table S2), although the c.1767C>T mutation in *JAK3* passes the 1.2 threshold, which could be considered the best candidate for affecting gene splicing in the absence of additional information. However, Skippy also integrates the MaxEntScan algorithm for evaluating the strength of splice sites and can automatically indicate potential activation of cryptic splice sites in the mutation neighborhood (Figure S1F).

SPANR (http://tools.genes.toronto.edu) uses DNA sequence features extracted from the neighborhood of more than 10,000 exons that show evidence of alternative splicing, and RNA-Seq data from the Illumina Body Map 2.0 project to estimate the percent of transcripts with the exon of interest spliced in (PSI) (S8). Unlike other tools, SPANR can evaluate the impact of mutations that are located as far as 300 bps away from an exon, but it does not evaluate the possibility of cryptic splice site activation. For a given mutation, the tool estimates the change in PSI across 16 tissue types and reports the biggest estimated change across all the tissues (Table S2). According to values produced for the three synonymous mutations of interest, none of them appear as strong candidates for experimental validation of splicing alterations as they all are predicted to have minor impact.

2 **Supplementary Table 1.** Hematological reconstitution of the patient after HSCT

**Days post-transplant**	**Autologous cells**	**Leucocyte/µL**	**PMN/µL**	**Lymphocyte/µL**
+15	3%	900	570	120
+32	2%	6000	4640	530
+61	1%	1900	1370	280
+91	0	4600	3090	730
+6 months	0	16420	3120	2090
+9 months	0	7200	2670	1490
+12 months	0	5600	2970	1320

3 **Supplementary Table 2**. Computational predictions of the impact on gene splicing of four synonymous mutations using Skippy and SPANR tools.

Mutation	Gene	Coordinate (hg19)	Skippy LOR	SPANR dPSI
c.333T>A	IL7R	chr5:35867519.T>A	0.052	-0.46
c.1767C>T	JAK3	chr19:17947957.G>A	1.29	-0.06
c.2961C>T	JAK3	chr19:17942054.G>A	-0.568	-0.20
c.333T>C	IL7R	chr5:35867519.T>C	0.394	+0.09

3 Supplementary Figures

A

Variant and Exon Summaries									
Variant Information				Exon(s) in Which the Variant is Located					
Variant No.	Var Chr.	Variant Position	Variant	Gene	Ensembl Exon Id	Exon Start	Exon End	Strand	Exon Length
1	chr5	35867519	T->A	IL7R	ENSE00001130282	35867408	35867565	+	158
2	chr19	17947957	C->T	JAK3	ENSE00000689815	17947938	17948022	-	85
3	chr19	17942054	C->T	JAK3	ENSE00000689798	17942037	17942209	-	173

B

Changes in Exonic Splicing Regulatory Elements (ESRs) [Help]							
	Changes in ESRs [Help]			Breakdown of Specific ESR Changes [Help]			
Variant No.	No. of ESE Losses	No. of ESE Gains	No. of ESS Gains	ESE→neutral	neutral→ESS	ESE→ESS	Log Odds Ratio Total [Help]
1	0	0	1	0	1	0	0.052
2	0	0	2	0	2	0	1.29
3	1	0	0	1	0	0	-0.568

C

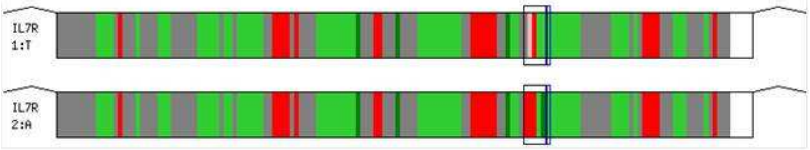

D

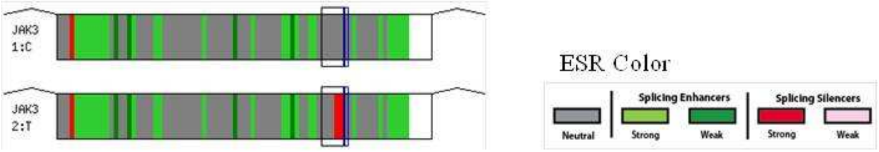

E

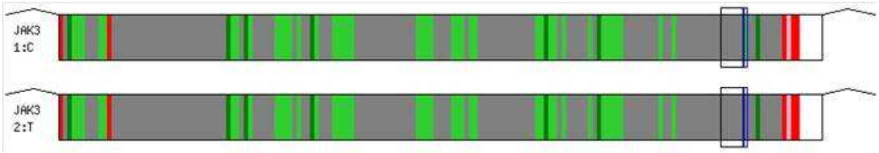

F

Exonic Environment [Help]							
		Splice Junction Strengths [Help]		Change in Splice Site (SS) Score Surrounding Variant [Help]			
Variant No.	Ensembl Exon Id	MaxEnt 5' Splice Site Score	MaxEnt 3' Splice Site Score	max(Δ3'SS)	MaxEnt Score of Mutant 3' SS	max(Δ5'SS)	MaxEnt Score of Mutant 5' SS
1	ENSE00001130282	9.14	9.26	0	0	3.81	9.45
2	ENSE00000689815	3.45	8.50	0	0	7.75	6.96
3	ENSE00000689798	3.44	9.26	0	0	7.76	7.41

Supplementary Figure 1. Select graphical output produced by the Skippy tool in the analysis of three synonymous mutations.
(A) Summary mutation information. **(B)** Detailed information on changes in exonic splicing regulatory elements (ESRs) induced by each mutation and corresponding LOR score assigned. **(C)** – **(E)** Graphical representation of the changes in ESRs in the affected exon. The specific regions affected by each mutation are denoted by blue rectangles. **(F)** Scores of canonical splice sites and changes in scores for cryptic splice sites as computed with the integrated MaxEntScan algorithm.

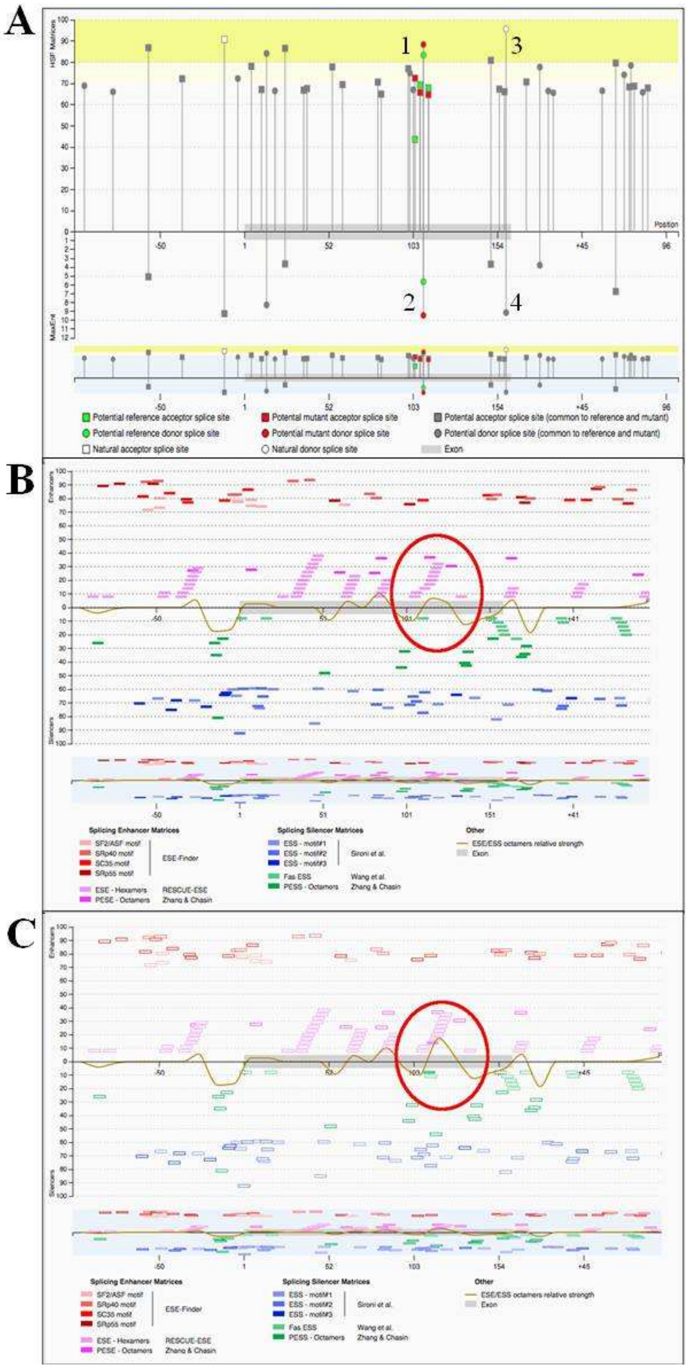

Supplementary Figure 2. Graphic output produced by the Human Splicing Finder tool for the analysis of the c.333T>A mutation in *IL7R*

(A) Cryptic donor splice site with GT dinucleotide at position c.331 is predicted to be enhanced by the mutation with **(1)** a score of 88.37 instead of 83.35 for HSF matrix, **(2)** a score of 9.45 instead of 5.64 for MaxEntScan, while natural donor site is predicted with **(3)** a score of 95.68 for HSF matrix and **(4)** 9.14 for MaxEntScan. **(B)** ESE/ESS ratio (golden curve) for WT sequence shows a low positive value for c.333T, whereas **(C)** the same ratio reaches a higher peak for c.333A in *IL7R* gene.

SUPPLEMENTARY REFERENCES

S1. Mueller WF, Hertel KJ (2012) RNA elements involved in splicing. In S. Stamm, C. Smith, R. Lührmann (Eds.), *Alternative pre-mRNA Splicing. Theory and Protocols* (pp. 23-31). Weinheim, Germany: Wiley-VCH Verlag & Co.

S2. Dogan RI, Getoor L, Wilbur WJ, Mount SM. SplicePort--an interactive splice-site analysis tool. *Nucleic Acids Res* (2007) 35:285-91.

S3. Desmet FO, Hamroun D, Lalande M, Collod-Béroud G, Claustres M, Béroud C. Human Splicing Finder: an online bioinformatics tool to predict splicing signals. Nucleic Acids Res (2009) 37(9):e67. doi: 10.1093/nar/gkp215.

S4. Yeo G, Burge CB. (2004) Maximum entropy modeling of short sequence motifs with applications to RNA splicing signals. *J Comput Biol*. 11(2-3):377-94.

S5. Reese MG, Eeckman FH, Kulp D, Haussler D. (1997) Improved splice site detection in Genie. *J Comp Biol*. 4(3):311-23.

S6. Brunak S, Engelbrecht I, Knudsen S. (1991) Prediction of human mRNA donor and acceptor sites from the DNA sequence. *J Mol Biol*. 220:49-65.

S7. Woolfe A, Mullikin J, and Elnitski L. Genomic features defining exonic variants that modulate splicing. Genome Biol (2010) 11(2):R20. doi:10.1186/gb-2010-11-2-r20.

S8. Xiong HY, Alipanahi B, Lee LJ, Bretschneider H, Merico D, Yuen RK, Hua Y, Gueroussov S, Najafabadi HS, Hughes TR, Morris Q, Barash Y, Krainer AR, Jojic N, Scherer SW, Blencowe BJ, Frey BJ. RNA splicing. The human splicing code reveals new insights into the genetic determinants of disease. *Science* (2015) 347(6218):1254806. doi: 10.1126/science.1254806.
